# Systematic review and meta-analysis of the association between *ABCA7* common variants and Alzheimer’s disease in non-Hispanic White and Asian cohorts

**DOI:** 10.3389/fnagi.2024.1406573

**Published:** 2024-10-17

**Authors:** Da Liu, Hongwei Zhang, Cao Liu, Jianyu Liu, Yan Liu, Na Bai, Qiang Zhou, Zhiyao Xu, Linyan Li, Hua Liu

**Affiliations:** ^1^Department of Clinical Medicine, North Sichuan Medical College, Nanchong, China; ^2^Department of Neurology, The Third People's Hospital of Chengdu, Chengdu, China; ^3^Chengdu Municipal Health Commission, Chengdu, China; ^4^Department of Neurology, The Sixth People’s Hospital of Chengdu, Chengdu, China; ^5^Medical College of Southwest Jiaotong University, Chengdu, China

**Keywords:** Alzheimer’s disease, *ABCA7*, SNPs, meta-analysis, systematic review

## Abstract

**Background and aims:**

The relationship between the *ABCA7* gene and Alzheimer’s disease (AD) has been widely studied across various populations. However, the results have been inconsistent. This meta-analysis aimed to evaluate the association of *ABCA7* polymorphisms with AD risk, including specific subtypes such as late-onset Alzheimer’s disease (LOAD).

**Methods:**

Relevant studies were identified through comprehensive database searches, and the quality of each study was assessed using the Newcastle-Ottawa Scale (NOS). Allele and genotype frequencies were extracted from the included studies. The pooled odds ratios (OR) with corresponding 95% confidence intervals (CI) were calculated using random-effects or fixed-effects models. Multiple testing corrections were conducted using the false discovery rate (FDR) method. The Cochran Q statistic and I^2^ metric were used to evaluate heterogeneity between studies, while Egger’s test and funnel plots were employed to assess publication bias.

**Results:**

A total of 36 studies, covering 21 polymorphisms and involving 31,809 AD cases and 44,994 controls, were included in this meta-analysis. NOS scores ranged from 7 to 9, indicating high-quality studies. A total of 11 SNPs (rs3764650, rs3752246, rs4147929, rs3752232, rs3752243, rs3764645, rs4147934, rs200538373, rs4147914, rs4147915, and rs115550680) in *ABCA7* were significantly associated with AD risk. Among these SNPs, two (rs3764650 and rs3752246) were also found to be related to the late-onset AD (LOAD) subtype. In addition, two SNPs (rs4147929 and rs4147934) were associated with the susceptibility to AD only in non-Hispanic White populations. A total of 10 SNPs (rs3764647, rs3752229, rs3752237, rs4147932, rs113809142, rs3745842, rs3752239, rs4147918, rs74176364, and rs117187003) showed no significant relationship with AD risk. Sensitivity analyses confirmed the reliability of the original results, and heterogeneity was largely attributed to deviations from Hardy–Weinberg equilibrium, ethnicity, and variations between individual studies.

**Conclusion:**

The available evidence suggests that specific *ABCA7* SNPs may be associated with AD risk. Future studies with larger sample sizes will be necessary to confirm these results.

**Systematic review registration:**

https://www.crd.york.ac.uk/prospero/, identifier: CRD42024540539.

## 1 Introduction

Alzheimer’s disease (AD), a neurological condition observed predominantly in the elderly population, is characterized by the presence of memory disorder, cognitive impairment, and loss of autonomy. Currently, there are 55 million individuals suffering from AD or other types of dementia, with an estimated global increase to 139 million by the year 2050 (Reitz et al., 2023). AD is a multifaceted disorder influenced by both genetic (approximately 70%) and environmental factors (approximately 30%) (Ferrari and Sorbi, 2021; Dorszewska et al., 2016). Consequently, some studies have estimated the relative importance of genetic susceptibility in Alzheimer’s disease.

ATP-binding cassette subfamily A member 7 (*ABCA7*) is a member of a subfamily that exhibits significant expression in the brain, particularly in neurons and microglia (Kim et al., 2006). *ABCA7* exhibits considerable similarity to the cholesterol-efflux transporter ABCA1 and participates in the cellular transport of lipids (Meurs et al., 2012). Furthermore, *ABCA7* plays a role in modulating the phagocytosis of apoptotic cells by macrophages (Jehle et al., 2006). Research also indicates that *ABCA7* is implicated in the pathogenesis of AD by influencing the processing of amyloid precursor protein, thereby reducing the production of amyloid beta (Aβ) (Sakae et al., 2016). Notably, genome-wide association studies (GWAS) revealed a significant correlation between the *ABCA7* gene and AD. To date, numerous studies have been carried out to explore the correlation between *ABCA7* gene polymorphisms and the risk of AD. However, the conclusions have been inconsistent, which might be due to factors such as small sample sizes, ethnic differences, and methodological differences. For example, contradictory conclusions were observed in two studies, one involving Asians (Liao et al., 2014) and the other involving non-Hispanic Whites (NHWs) (Öznur et al., 2015), likely resulting from racial differences. Genetic variations in the *ABCA7* gene may exhibit differences in different populations, affecting *ABCA7* gene expression and protein function, leading to differences in susceptibility to AD among different ethnic groups. Even individuals with similar variations may exhibit different phenotypes due to differences in genetic backgrounds and environmental and other genetic factors. Previous studies have predominantly focused on specific populations, with limited exploration of other genetic backgrounds, thereby constraining a comprehensive understanding of the association between *ABCA7* and AD. Therefore, exploring the differential impact of *ABCA7* on AD among different races is essential, as it can contribute to personalized medical treatments and precise risk assessments. Two other studies involving Asian populations also yielded inconsistent results, possibly due to different sample sizes (Miyashita et al., 2013; Yamazaki et al., 2017). Specifically, Miyashita et al. conducted a study with a larger sample size (involving 1,008 AD patients and 1,016 controls), while Yamazaki et al. conducted a study with a smaller sample size (involving 50 AD patients and 50 controls). Additionally, Cascorbi et al.’s (2013) study demonstrated evidence of the relationship between *ABCA7* and AD in NHWs, but this significant association was not successfully replicated in another NHW study (Omoumi et al., 2014). The discrepancy in results from the two studies may be attributed to differences in sample selection and SNP genotyping methods.

A meta-analysis is often used to detect and validate minimal biological effects in genetic association studies of complex diseases (Munafò and Flint, 2004). Using meta-analyses, researchers have investigated the role of a few single-nucleotide polymorphisms (SNPs) on the *ABCA7* locus in AD across different populations. However, the latest meta-analysis was published in 2019 (De Roeck et al., 2019). Multiple new studies on the relationship between the *ABCA7* gene and AD have been published in recent years. In addition, the association of other genetic variants and SNPs in the *ABCA7* gene with AD deserves further analysis. A meta-analysis, encompassing all available studies, was conducted to establish statistical support for an association between *ABCA7* gene polymorphisms and susceptibility to AD.

## 2 Methods

### 2.1 Study design

The protocol of the present systematic review and meta-analysis was registered in the international Prospective Register of Systematic Reviews (PROSPERO) database (registration number CRD42024540539). To conduct this systematic review and meta-analysis, we followed the Preferred Reporting Items for Systematic reviews and Meta-Analyses statements guidelines (Page et al., 2021). *ABCA7* gene polymorphisms were used as the exposure and AD was used as an outcome. This study did not require the approval of an ethics committee.

### 2.2 Data collection

Three investigators (Liu D, Zhang H, and Bai N) independently identified all studies that explored the correlation between *ABCA7* gene polymorphisms and AD by searching the following databases until November 2023: PubMed (from 1966), EMBASE (from 1966), the Cochrane Library (from 2003), ProQuest Dissertations & Theses Database (from 1980), Web of Science (from 1990), China National Knowledge Infrastructure (CNKI) (from 1994), and Wanfang Database (including journal articles, dissertations or theses, and conference literature, from 1990). We used the following keywords or a combination of them in the search strategy: “Alzheimer’s disease,” “AD,” or “dementia,” and “*ABCA7*,” or “ATP Binding Cassette Subfamily A Member 7.” This research is limited to human studies. We further searched for possible eligible studies in the references within the retrieved articles, review articles, and abstracts from recent conferences.

Only the most recent or complete reports were selected for analysis if the same or a similar patient cohort was included in several publications. Studies that met the following criteria were included for meta-analysis: (1) The association between *ABCA7* gene variants and AD was examined by using a population-based, case–control, or cohort design; (2) AD was diagnosed using a widely accepted standard; (3) control subjects were unrelated individuals with no dementia confirmed by physicians; (4) genotype or allele frequencies were reported directly or could be calculated in both AD cases and controls, or studies provided directly with *p*-values, odds ratio (OR) values, and 95% confidence intervals (95%CI); (5) a genetic variant of the *ABCA7* gene had been included in at least two studies (Liu et al., 2017).

### 2.3 Data extraction

Data were carefully extracted from all eligible studies independently by three authors (Zhou Q, Xu Z, and Li L), and any disagreements were resolved through discussion. The following information was extracted: first author’s surname, year of publication, country of origin, study design, sex composition of the case and control groups, ethnicity of the subjects studied, total number of subjects, definition and characteristics of cases and controls, genetic variants associated with AD, genotyping methods, distribution of genotypes and alleles, AD subtype (if reported), information on additional genetic variants, as well as gene–gene and gene–environment interactions (if investigated). Genotype frequencies were calculated where possible. For studies that included subjects from different ethnic groups, the data were extracted separately for each ethnic group. When some of the information was unavailable, we emailed the corresponding author for additional data.

### 2.4 Data analysis and statistical analyses

The quality of the included studies was assessed using the Newcastle-Ottawa Scale (NOS). An NOS score of ≥7 was considered to indicate high quality (Bai et al., 2022).

ORs and pooled ORs with corresponding 95%CI were calculated using fixed-effects and random-effects models (Borenstein et al., 2010). A chi-squared test based on the Cochran Q statistic (*p*-values<0.10 being considered statistically significant) was used to evaluate the heterogeneity between studies (Kulinskaya and Dollinger, 2015), and the I^2^ index was used to quantify the heterogeneity (Higgins et al., 2002).

Hardy–Weinberg equilibrium (HWE) was tested using the chi-squared test in the control groups (Bosco et al., 2012). The meta-analysis examined the relationship between each polymorphism and the AD risk through three genetic comparisons: (1) allelic comparison (AC), (2) dominant model (DM), and (3) recessive model (RM) (Zintzaras and Lau, 2008). Only when both alleles and genotype distribution were available did multiple testing corrections be conducted using the false discovery rate (FDR) method proposed by Benjamini and Hochberg (Parks et al., 2018; Chen, 2020). A funnel plot was employed to investigate potential publication bias in analyses involving different polymorphisms, and asymmetries were considered if the Egger’s test has a *p-value* of <0.10 (Egger et al., 1997).

The LDpop tool^1^ was utilized to query linkage disequilibrium (LD) in genomic datasets from various reference populations. D′ ranges from 0 to 1, with 0 indicating no linkage and 1 indicating perfect linkage between 2 markers. In contrast to R^2^, D′ is not influenced by differences in allele frequencies between ethnic groups (Reitz et al., 2013).

Sub-population analyses were conducted for ethnicity (Zintzaras and Lau, 2008), and subgroup analyses for AD subtypes, AD sample source, family history, or sex were also performed. Sensitivity analyses were performed after excluding specific studies (Zintzaras and Lau, 2008), such as studies in which populations in the control group did not conform to HWE distribution, studies with 0 genotype distribution in each study group, and studies with mixed data (from neuropathological and clinical diagnosis). All statistical analyses were performed with the Cochrane Review Manager (version 5.3) and STATA 17.0 package. A probability value of *p* < 0.05 (two-tailed) was considered significant unless indicated otherwise.

## 3 Results

### 3.1 Eligible studies and study characteristics

**Figure 1:**
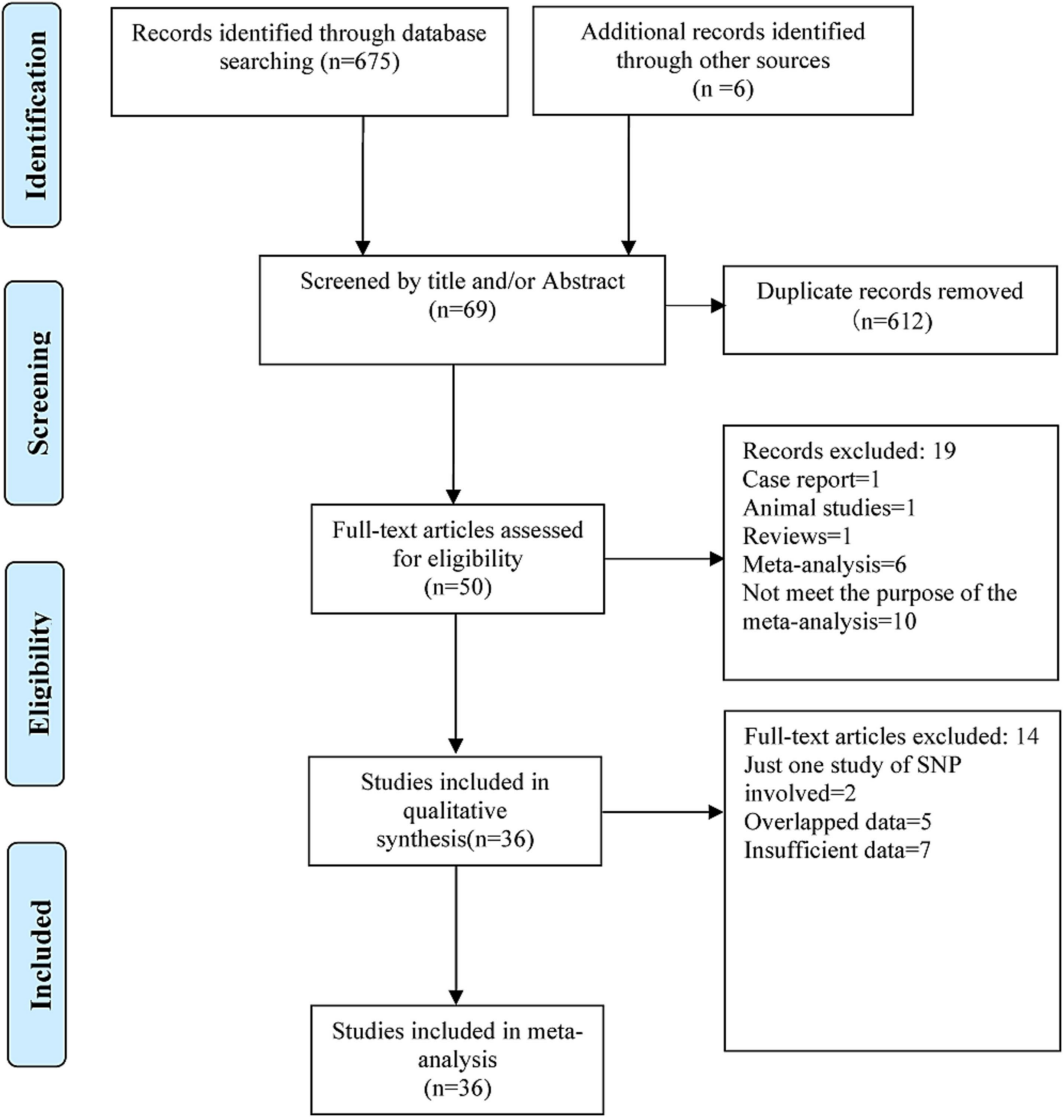
Preferred reporting items for systematic reviews and meta-analysis (PRISMA) flow chart for literature search and study selection in the present meta-analysis.

The search results showed that there were 91 items in the Chinese database, including 29 in CNKI and 62 in the Wanfang Database, and 584 items from the English database, including 261 in Pubmed, 36 in Embase, 2 in the Cochrane Library, 276 in Web of Science, as well as 9 in the ProQuest Dissertations & Theses Database. In addition, six items were screened out of the reference lists of the meta-analysis articles retrieved above. A total of 681 items were retrieved. Initially, 612 records were excluded because duplicate items were included across databases. Furthermore, 19 items were excluded after reading the titles and abstracts as they were not relevant. Finally, after reading the full text, 50 potentially relevant articles were initially selected for this meta-analysis. Of which, 36 articles (Harold et al., 2009; Hollingworth et al., 2011; Logue et al., 2011; Naj et al., 2011; Cascorbi et al., 2013; Chung et al., 2013; Lambert et al., 2013; Miyashita et al., 2013; Reitz et al., 2013; Tan et al., 2013; Liao et al., 2014; Liu et al., 2014b; Omoumi et al., 2014; Cuyvers et al., 2015; Öznur et al., 2015; Steinberg et al., 2015; Cukier et al., 2016; Sassi et al., 2016; Dos Santos et al., 2017; Li et al., 2017; Moreno et al., 2017; Yamazaki et al., 2017; Kjeldsen et al., 2018; Moreno-Grau et al., 2018; Patel et al., 2018; Liu, 2018; Zhang et al., 2018; Fehér et al., 2019; Abd Elrahman et al., 2020; Talebi et al., 2020; Nazaketi et al., 2020; Ren, 2020; Hou et al., 2021; Campbell et al., 2022; Jiao et al., 2022; Wang et al., 2022) were considered to be eligible for the final meta-analysis after removing 14 of them because of duplication or insufficient data. The results of the systematic literature search and article selection are summarized in Figure 1. The removed articles and the reasons for excluding each article are given in Supplementary Table S1.

Only SNP variation in the *ABCA7* gene is eligible for the meta-analysis in these studies. Standard methods such as polymerase chain reaction, enzyme digestion, and gel electrophoresis or sequencing were used to identify genotypes of these SNPs. The NOS scores of these studies ranged from 7 to 9, suggesting that the methodological quality of all studies was acceptable. The characteristics of the studies included, as well as the 21 *ABCA7* SNPs involved, are summarized in Supplementary Table S2.

### 3.2 Genetic association of *ABCA7* SNPs with AD

#### 3.2.1 SNP rs3764650

The association of rs3764650 with AD risk was investigated in 25 studies (Harold et al., 2009; Hollingworth et al., 2011; Logue et al., 2011; Naj et al., 2011; Cascorbi et al., 2013; Chung et al., 2013; Miyashita et al., 2013; Tan et al., 2013; Liao et al., 2014; Liu et al., 2014b; Omoumi et al., 2014; Cuyvers et al., 2015; Öznur et al., 2015; Dos Santos et al., 2017; Li et al., 2017; Moreno et al., 2017; Yamazaki et al., 2017; Liu, 2018; Zhang et al., 2018; Abd Elrahman et al., 2020; Talebi et al., 2020; Nazaketi et al., 2020; Ren, 2020; Hou et al., 2021; Wang et al., 2022), involving 31,809 cases and 44,994 controls.

**Figure 2:**
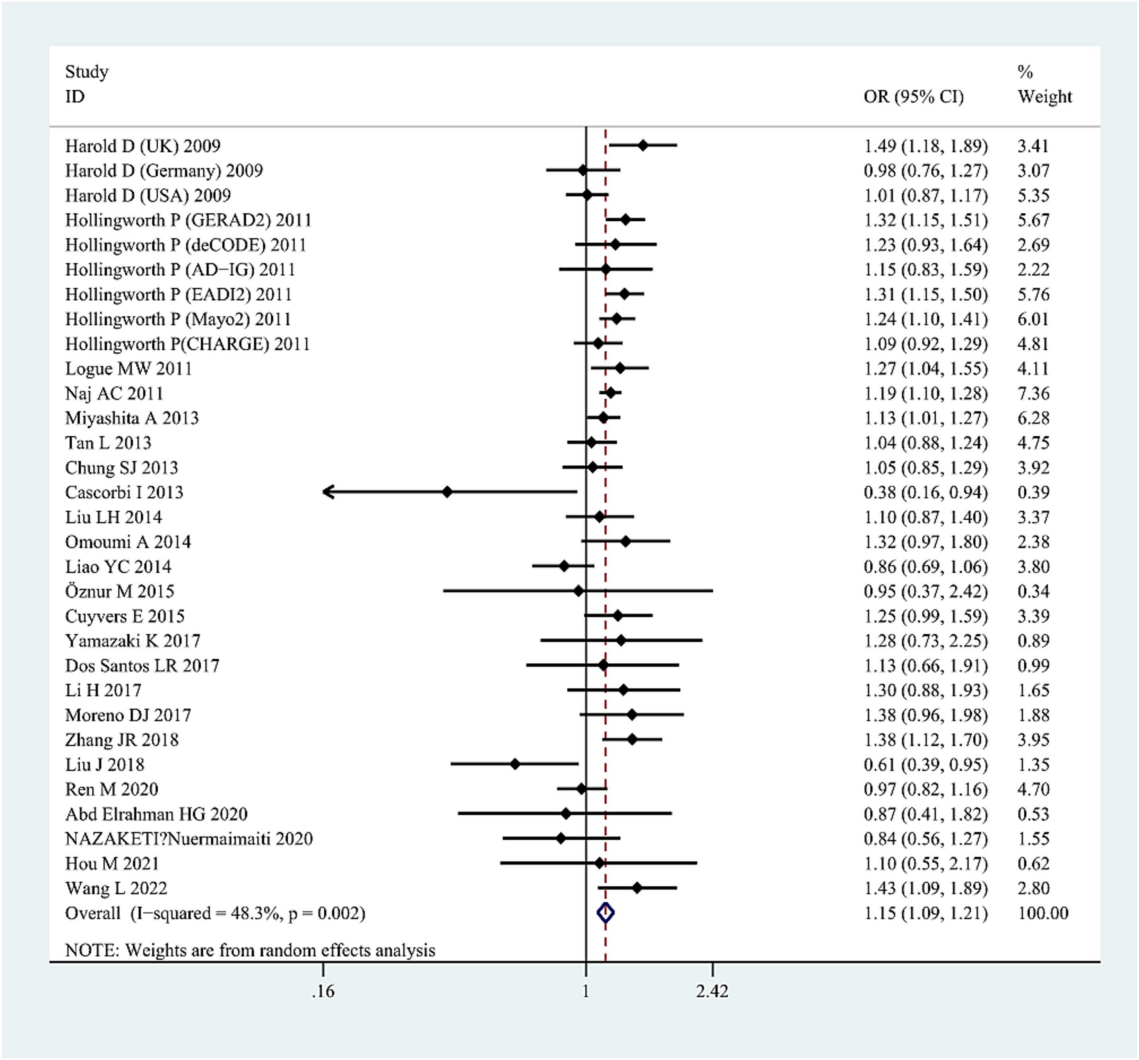
Forest plot of *ABCA7* rs3764650 allelic comparison (G vs. T) and AD susceptibility in combined population. Horizontal lines are 95% confidence intervals (CI). The contrast has an OR of 1.15 (95%CI: 1.09–1.21, *p* < 0.0001) in the random-effects model.

**Figure 3:**
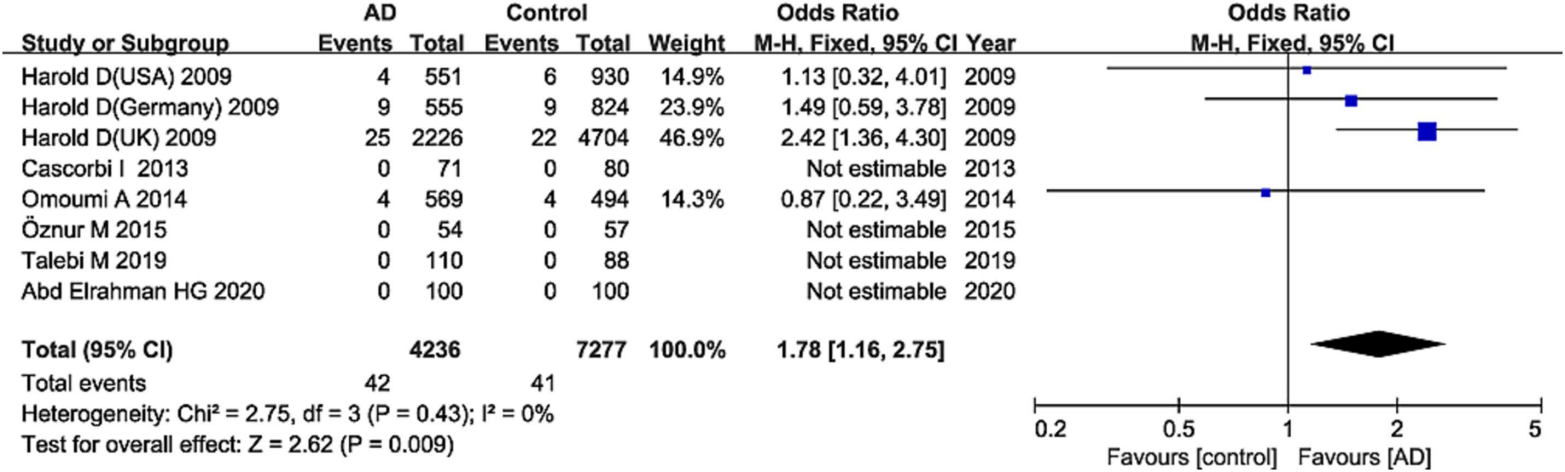
Forest plot of *ABCA7* rs3764650 recessive model (GG vs. (TT + TG)) in the NHW population. The contrast has an OR of 1.78 (95% CI: 1.16–2.75, *p* = 0.009) in the fixed-effects model.

The G allele was found to have a significant relationship to AD risk in the combined population (OR = 1.15, 95%CI: 1.09–1.21, FDR-corrected *P* (*P*_FDR_) = 0.0003) (Figure 2; Supplementary Table S3), the Asian studies (OR = 1.10, 95% CI: 1.03–1.17, *P*_FDR_ = 0.009), the NHW studies (OR = 1.19, 95% CI: 1.11–1.28, *P*_FDR_ = 0.0003), and the late-onset AD (LOAD) subtype (OR = 1.17, 95% CI: 1.11–1.23, *P*_FDR_ = 0.0003). The GG genotype increased the susceptibility to AD only in NHWs (OR = 1.78, 95%CI: 1.16–2.75, *P*_FDR_ = 0.0135) (Figure 3; Supplementary Table S3).

**Figure 4:**
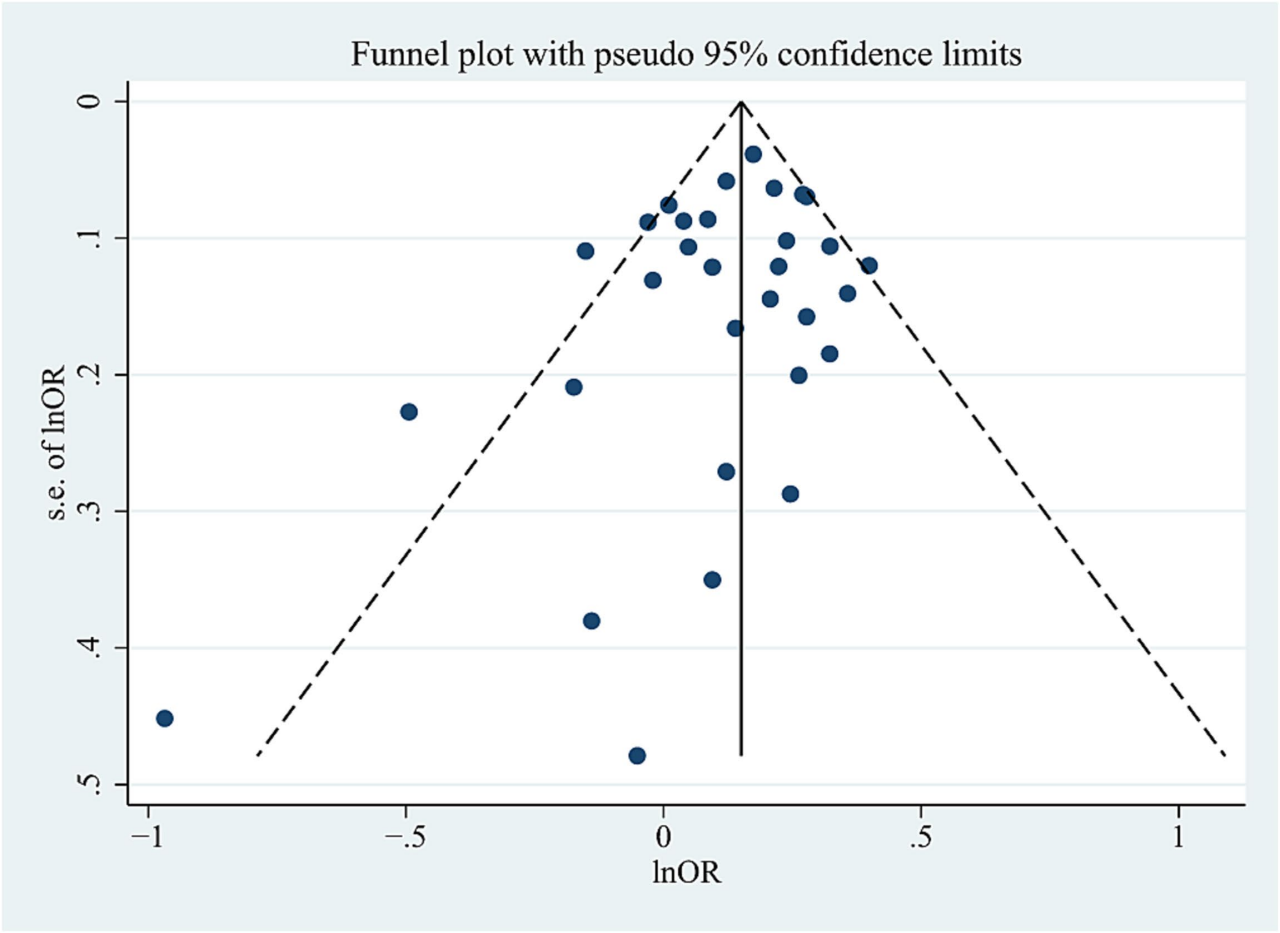
Funnel plot of *ABCA7* rs3764650 allele distribution in the allelic comparison in combined population.

Significant heterogeneity was detected in the combined population (AC: I^2^ = 45%, *p* = 0.006; DM: I^2^ = 48%, *p* = 0.01; RM: I^2^ = 63%, *p* = 0.00005); however, the heterogeneity disappeared when the Asian studies were excluded, suggesting that ethnicity (Asians) may be the source of heterogeneity. High heterogeneity was also detected in NHW studies (AC: I^2^ = 41.6%, *p* = 0.046; DM: I^2^ = 63%, *p* = 0.04). After excluding studies with neuropathological diagnosis (Cascorbi et al., 2013), heterogeneity disappeared, suggesting that heterogeneity may be caused by differences in study subjects. The sensitivity analysis excluded specific studies successively, such as studies in which populations in the control group did not conform to HWE distribution (Harold et al., 2009; Hollingworth et al., 2011; Logue et al., 2011; Liao et al., 2014; Cuyvers et al., 2015; Li et al., 2017; Talebi et al., 2020), studies with 0 genotype distribution in each study group (Cascorbi et al., 2013; Öznur et al., 2015; Talebi et al., 2020; Hou et al., 2021), as well as studies with all samples from neuropathological diagnosis (Cascorbi et al., 2013), did not alter the final results in AC and DM in the combined or subgroup populations, further confirming the reliability of the results. However, the sensitivity analyses (excluding one study with neuropathological diagnosis [Cascorbi et al., 2013]) did not maintain the results of the RM in the NHW or Asian populations. We did not find publication bias for this SNP in the combined population using the funnel plots and Egger’s test (*p* = 0.171 in the AC model) (Figure 4).

#### 3.2.2 SNP rs3752246

Eleven articles (Logue et al., 2011; Naj et al., 2011; Tan et al., 2013; Cuyvers et al., 2015; Sassi et al., 2016; Moreno et al., 2017; Patel et al., 2018; Liu, 2018; Zhang et al., 2018; Fehér et al., 2019; Jiao et al., 2022) explored the relationship of SNP rs3752246 (15,834 cases and 16,526 controls) to AD.

**Figure 5:**
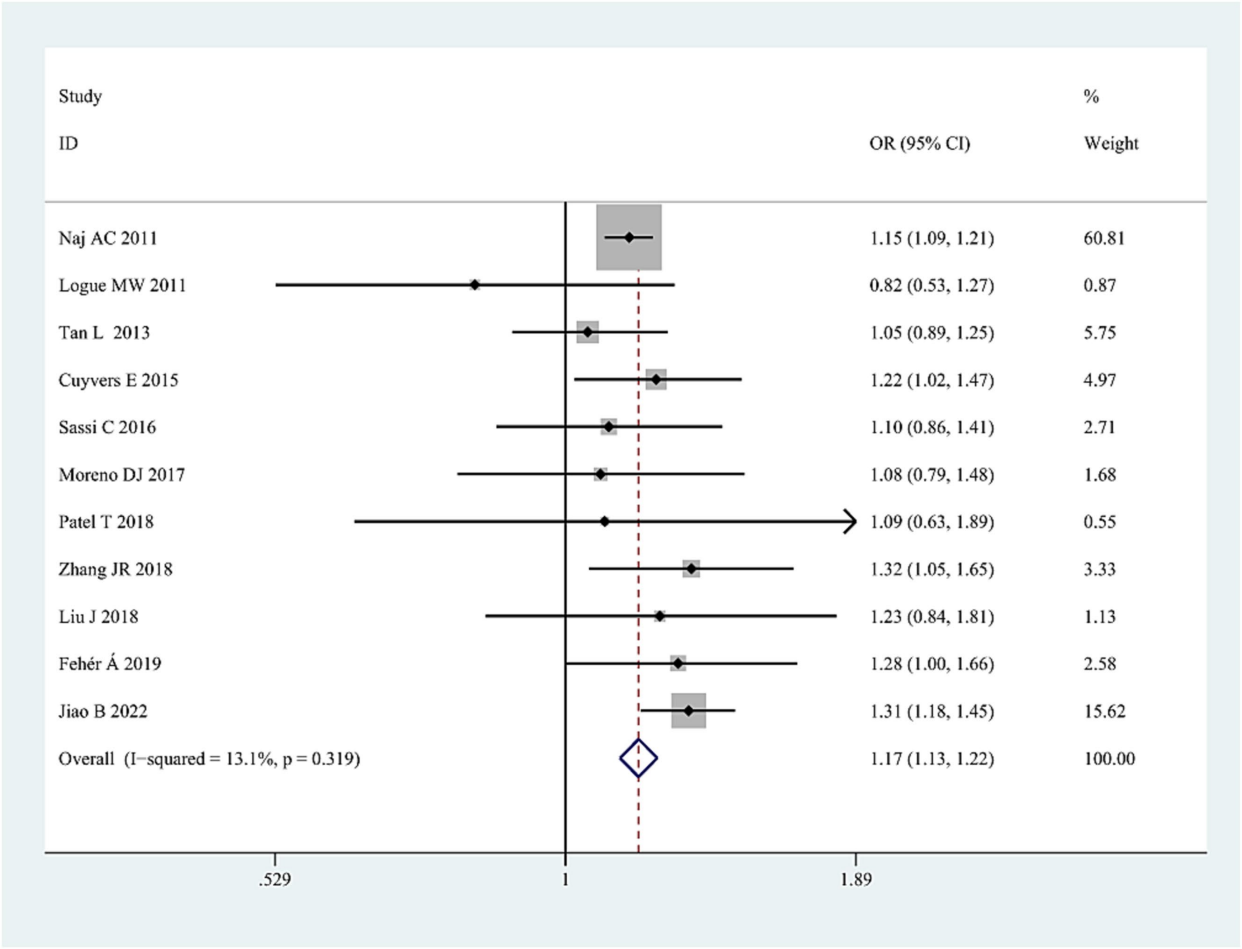
Forest plot of *ABCA7* rs3752246 allelic comparison (G vs. C) and AD susceptibility in combined population. The contrast has an OR of 1.17 (95% CI: 1.13–1.22, *p* < 0.0001) in the fixed-effects model.

The pooled results showed that the G allele was associated with AD risk in the combined population (OR = 1.17, 95%CI: 1.13–1.22, *P*_FDR_ = 0.0003) (Figure 5; Supplementary Table S3), the Asian studies (OR = 1.24, 95%CI: 1.14–1.35, *P*_FDR_ = 0.0003), the NHW studies (OR = 1.16, 95% CI: 1.10–1.22, *P*_FDR_ = 0.0003), and the LOAD subtype (OR = 1.15, 95% CI: 1.10–1.21, *P*_FDR_ = 0.0003). No significant association was found in other genetic comparisons in any population.

Heterogeneity among studies was detected only in RM (GG/(CC + CG)) in the combined population (I^2^ = 71%, *p* = 0.02). The heterogeneity disappeared after excluding the study by Sassi et al. (2016) for unknown reasons, suggesting that sometimes individual studies may cause heterogeneity. The sensitivity analysis, excluding specific studies successively (studies with deviation from HWE in controls (Logue et al., 2011; Cuyvers et al., 2015; Sassi et al., 2016; Liu, 2018)) and studies with all samples from neuropathological diagnosis (Patel et al., 2018), did not alter the final results, further confirming the reliability of the results. No significant publication bias was detected in all three genetic models.

#### 3.2.3 SNP rs4147929

The role of rs4147929 in AD was analyzed in 8 studies (Lambert et al., 2013; Cuyvers et al., 2015; Kjeldsen et al., 2018; Moreno-Grau et al., 2018; Liu, 2018; Talebi et al., 2020; Ren, 2020; Wang et al., 2022) involving 13,025 cases and 117,807 controls.

The A allele increased the susceptibility to AD in the combined population (OR = 1.11, 95%CI: 1.02–1.22, *p* = 0.017), in the Asians (OR = 1.21, 95%CI: 1.00–1.21, *p* = 0.0049), and in NHWs (OR = 1.16, 95%CI: 1.11–1.21, *p* < 0.0001). However, only one positive result passed FDR adjustment: the NHW population (A allele: OR = 1.16, 95%CI: 1.11–1.21, *P*_FDR_ = 0.0003) (Supplementary Table S3). There was no significant association with AD in other genetic comparisons in all populations.

Heterogeneity with statistical significance was found in the combined population (AC: I^2^ = 63%, *p* = 0.009; DM: I^2^ = 64%, *P*_FDR_ = 0.02; RM: I^2^ = 94%, *P*_FDR_ < 0.00001), which disappeared after several studies were excluded in turn (Kjeldsen et al., 2018; Liu, 2018; Talebi et al., 2020; Ren, 2020; Wang et al., 2022). However, no specific cause for the heterogeneity was found.

The sensitivity analyses excluding the studies with HWE-deviated cohorts (Cuyvers et al., 2015; Talebi et al., 2020) did not affect the stability of the original results. We did not find publication bias for this SNP using the funnel plots or Egger’s test.

#### 3.2.4 SNP rs3764647, rs3752232, rs3764645, and rs4147934

For each of the four SNPs (rs3764647, rs3752232, rs3764645, and rs4147934), four studies analyzed their relationship with AD involving 1,943 to 2,424 cases and 3,636 to 3,898 controls. Since the original genotype distribution data were not obtained, we only performed an allelic comparison.

For rs3764647, no significant association with AD was observed in all populations. However, the G allele of rs3752232 played a protective role in AD in the combined population and Asians (Combined population: OR = 0.82, 95%CI: 0.70–0.95, *p* = 0.01; Asians: OR = 0.82, 95%CI: 0.69–0.96, *p* = 0.01) (Supplementary Table S3) (Figs not shown), the A allele of rs3764645 reduced risk for AD in the combined population (OR = 0.85, 95%CI: 0.79–0.92, *p* < 0.0001) (Supplementary Table S3) (Fig omitted), and the G allele of rs4147934 increased the susceptibility to AD in the NHW population (OR = 1.21, 95%CI: 1.07–1.37, *p* = 0.002) (Supplementary Table S3) (Fig omitted).

Significant heterogeneity was found among studies involving rs3764647 and rs4147934 (I^2^ = 81.1%, *p* = 0.001, I^2^ = 87%, *p* < 0.0001, respectively), which disappeared after removing the studies with deviation from HWE in controls (Logue et al., 2011; Cuyvers et al., 2015; Sassi et al., 2016), suggesting that HWE may be a source of heterogeneity.

The sensitivity analyses after excluding the study with HWE-deviated cohorts (Logue et al., 2011; Cuyvers et al., 2015; Sassi et al., 2016) for rs3764647, rs3764645 and rs4147934 loci and all samples from neuropathological diagnosis (Patel et al., 2018) for the four SNPs did not alter the results, further confirming the reliability of the results. No significant publication bias was detected in all genetic comparisons for these loci.

#### 3.2.5 Other SNPs

For each of the remaining 14 SNPs (rs3752229, rs3752237, rs3752243, rs4147932, rs3745842, rs3752239, rs4147914, rs4147915, rs4147918, rs74176364, rs115550680, rs117187003, rs113809142, and rs200538373), only two or three studies analyzed their association with AD, involving a total of up to 4,848 cases and 7,161 controls.

Five SNPs showed significant association with AD risk in the AC model (rs3752243 G allele: OR = 0.84, 95%CI: 0.76–0.92, *p* = 0.0001; rs4147914 A allele: OR = 1.28, 95%CI: 1.17–1.40, *p* < 0.0001; rs4147915 A allele: OR = 0.85, 95%CI:0.77–0.94, *p* = 0.001; rs115550680 A allele: OR = 1.84, 95%CI: 1.55–2.17, *p* < 0.0001; rs200538373 C allele: OR = 1.71, 95%CI: 1.20–2.44, *p* = 0.003) (Supplementary Table S3) (Figs not shown).

Due to insufficient data, other genetic comparisons, heterogeneity between studies, subgroup analysis, sensitivity analysis, and publication bias were not explored.

#### 3.2.6 Linkage disequilibrium analysis

This study used the LDpop tool to analyze the LD between SNP pairs associated with AD. We identified significant LD (D′ > 0.8) between certain SNP pairs, as detailed in Supplementary Table S4.

## 4 Discussion

### 4.1 Relationship between *ABCA7* gene polymorphisms and AD

The meta-analysis results showed that 11 SNPs (rs3764650, rs3752246, rs4147929, rs3752232, rs3752243, rs3764645, rs4147934, rs200538373, rs4147914, rs4147915, and rs115550680) in the *ABCA7* gene were significantly associated with AD risk, and two of these SNPs (rs3764650 and rs3752246) were also found to be related to the LOAD subtype. In addition, two SNPs (rs4147929 and rs4147934) were associated with susceptibility to AD only in NHWs. The other ten SNPs (rs3764647, rs3752229, rs3752237, rs4147932, rs113809142, rs3745842, rs3752239, rs4147918, rs74176364, and rs117187003) showed no significant relationship with AD risk.

Several previous meta-analyses also focused on the association between *ABCA7* gene polymorphisms and AD. The study by Liu et al. (2014a) observed a significant association between rs3764650 and AD. The meta-analysis from Bao et al. (2016) included 17 original studies and showed a significant association of *ABCA7* rs3764650 with an increased risk of AD. Zhou et al.’s (2017) meta-analysis, including 10 eligible studies, found that *ABCA7* rs3764650 polymorphism was significantly associated with AD. However, a meta-analysis by Wang et al. (2018), which included 12 primary studies, showed variations in the relationship between rs3764650 and AD in different models or races. Additionally, in the meta-analysis from Ma et al. (2018), including 16 original studies, three common loci were confirmed to increase the risk of AD, but the associations varied among the different races. However, these meta-analyses have some limitations, including failing to include all eligible studies (Liu et al., 2014a; Bao et al., 2016; Zhou et al., 2017; Ma et al., 2018; Wang et al., 2018), not excluding the controls that deviate from HWE (Bao et al., 2016; Ma et al., 2018), and providing only allelic comparison patterns (Bao et al., 2016; Zhou et al., 2017). In contrast, our study included more comprehensive literature and gene polymorphisms. Moreover, our approach improved the rigor and robustness of the results, such as the utilization of three comparison models and FDR correction. The present meta-analysis contained 36 original studies (including 21 SNPs, involving a total of up to 31,809 cases and 44,994 controls) and provided more comprehensive analysis of the relationship between *ABCA7* gene polymorphisms and AD risk.

### 4.2 Possible mechanisms of *ABCA7* gene polymorphisms in AD

The neuropathological characteristics of AD are defined as abnormal aggregation of Aβ peptide in the brain parenchyma and neurofibrillary tangles composed of abnormal hyperphosphorylation of Tau protein in neurons (Grangeon et al., 2023). The *ABCA7* gene is on chromosome 19p13.3, the same chromosomal section as *APOE*, a gene that is well known to have a strong association with AD. *ABCA7* promotes the efflux of lipids from cells to apolipoproteins and can also regulate phagocytosis and modulate the processing of amyloid precursor protein to generate the AD Aβ peptide, which is considered an important link in the pathogenesis of AD (Jehle et al., 2006; Meurs et al., 2012; Sakae et al., 2016). The previous studies also found that *ABCA7* SNPs were associated with brain amyloidosis (Apostolova et al., 2018), changes in gray matter density (Stage et al., 2016), and Braak staging, a measure of neurofibrillary tangle development, which is associated with cognitive decline (Lyssenko and Praticò, 2021).

The previous studies found that the *ABCA7* gene rs3764650-G allele was associated with cortical and hippocampal atrophy, cognitive performance, and neuritic plaque burden (Shulman et al., 2013; Andrews et al., 2016; Ramirez et al., 2016). Rs3752246-C allele correlated with lower levels of CSF Aβ42, and the alternate allele of rs4147934 was related to lower levels of CSF p-tau (Dong et al., 2022). Rs115550680 might regulate the effects of methylation on cognition (Chaar et al., 2022). These findings further confirm the role of the *ABCA7* gene in the pathogenesis of AD.

Our results showed that two SNPs (rs4147929 and rs4147934) were associated with susceptibility to AD in NHWs alone. Due to variations in population-specific LD and allele frequencies, the impact of risk genes and alleles shared among different ethnicities may differ across distinct populations for AD. From studies in individuals of African and Hispanic ancestry, notable ancestry-related differences have been identified in the genetic architecture of AD (Reitz et al., 2023). For example, the effect of *APOE ε4* is weaker in African American and Hispanic populations. However, its effect is higher in East Asian populations (Miyashita et al., 2023). A previous study found that the genetic architecture of LOAD in African Americans differs from that in individuals of European ancestry (Logue et al., 2018). Another study found that the *ABCA7* gene showed nominal significance in Caribbean Hispanics but not in European families (Zhao et al., 2019). These findings indicated that the impact of genetic ancestry on the risk of AD associated with *ABCA7* variants was different. This supports the fact that the risk of AD inheritance is not always the same in different ethnic groups, reflecting the heterogeneity of AD gene inheritance.

Research has found that, in African Americans, the SNP in *ABCA7* (rs115550680) was in LD with two other *ABCA7* SNPs (rs3764650 and rs3752246) previously associated with LOAD in non-Hispanic Whites of European ancestry and showed the same direction of effect (Reitz et al., 2013). The study by Cukier et al. (2016) identified a 44 base pair frameshift deletion in *ABCA7* (rs142076058) that is in LD with rs115550680. These findings indicated that certain SNPs in *ABCA7* exhibited LD in different ethnic groups, which partially supported our LD findings. The presence of these linkage disequilibria may affect the accuracy of genetic risk assessment.

### 4.3 Publication bias

An important source of bias in the meta-analysis arises from the tendency of journals to publish studies with positive findings, potentially skewing the source material.

In our analysis, funnel plot asymmetry was not found for *ABCA7* gene polymorphisms, suggesting the absence of publication bias. However, it is important to note that funnel plot asymmetry can result from other factors, such as variations in methodological quality or simply a play of chance.

### 4.4 Heterogeneity

Significant heterogeneity was observed across studies in genetic comparisons for several *ABCA7* polymorphisms. Multiple reasons may result in heterogeneity in meta-analysis, such as the source of the samples, ethnicity, and the characteristics of the single study. In the present analyses, the deviation from HWE may be the main cause of heterogeneity, which decreased significantly or disappeared when the studies not in HWE were removed from the analyses in combined population or sub-population for rs3764650, rs4147934, rs3764647. Second, the ethnic differences could partly explain the heterogeneity in some comparison models. Heterogeneity disappeared when the Asian studies were excluded from the combined population for rs3764650. In addition, the heterogeneity disappeared when the study with subjects from neuropathological diagnosis was removed from the analyses in the NHW subgroup for rs3764650. It is worth noting that, sometimes, individual studies may cause heterogeneity. The observed heterogeneity could be attributable to differences in environmental factors, methodological factors in design, and how the studies were conducted. As mentioned above, the presence of heterogeneity calls for caution in interpreting the current meta-analysis findings.

### 4.5 NOS evaluation

Since all the included studies were non-randomized and had a cohort or case–control design, the NOS was used to judge study quality, as recommended by the Cochrane Collaboration (Chopra et al., 2013). No study had a score of NOS < 7 in original studies, indicating a higher quality of studies included in the present meta-analysis as a whole.

### 4.6 Limitations of this meta-analysis

Our meta-analysis is subject to several limitations that warrant consideration. First, a possible limitation is language bias. We only found studies regarding *ABCA7* gene polymorphisms and AD in Chinese and English. Some articles published in foreign languages might not appear in international journal databases and could be missed by our searches. This limitation might affect the generalizability of the results, as relevant research published in other languages could provide additional insights and data. Furthermore, the exclusion of unpublished articles may have led to publication bias. Additionally, the omission of certain GWAS studies could impact the validity and comprehensiveness of our findings. Importantly, the limited number of studies focusing on the *ABCA7* gene SNPs in specific populations, such as African Americans, precluded subgroup analyses for these groups. Incorporating additional ethnic groups in future research efforts would enhance the generalizability of the findings and provide a more comprehensive understanding of the genetic factors involved. Notably, the findings concerning the rs3752237, rs3752243, rs4147932, rs3745842, rs3752239, rs4147914, rs4147915, rs4147918, rs74176364, rs115550680, rs117187003, rs113809142, and rs200538373 polymorphisms are based on a limited number of studies, necessitating further confirmation and updates. Finally, significant heterogeneity was observed among the studies analyzing the rs3752246, rs3764650, rs4147929, rs3764647, and rs4147932 polymorphisms. Although some heterogeneity was mitigated by excluding specific studies, the underlying causes remain unclear. Consequently, the conclusions drawn from this meta-analysis should be interpreted with caution.

## 5 Conclusion

In conclusion, the evidence gathered thus far suggests that SNPs within the *ABCA7* gene are associated with an increased risk of AD. This meta-analysis included a larger number of original studies and used methods such as three comparison models and FDR correction to improve the rigor and robustness of the results. Notably, three common variants in *ABCA7*—rs3764650, rs3752246, and rs4147929—have consistently been identified as significantly associated with heightened AD risk, aligning with the findings of previous meta-analyses. Additionally, our analysis has identified several SNPs in the *ABCA7* gene—rs3752232, rs3752243, rs3764645, rs4147934, rs200538373, rs4147914, rs4147915, and rs115550680—which were unreported in previous meta-analyses and have now been identified as significantly associated with AD risk. Future studies should focus on validating these findings by improving methodological quality, increasing sample sizes, and incorporating more ethnically diverse populations.

## Data Availability

The original contributions presented in the study are included in the article/Supplementary material, further inquiries can be directed to the corresponding author.

## Footnotes

^1^ https://ldlink.nih.gov/?tab=ldpop
