# Supplementary material for: Systematic review and meta-analysis of the association between ABCA7 common variants and Alzheimer’s disease in non-Hispanic White and Asian cohorts
Source: Front Aging Neurosci. 2024 Oct 17;16:1406573. doi: 10.3389/fnagi.2024.1406573 (PMC11524920; doi:10.3389/fnagi.2024.1406573)
Supplement: Supplementary file 1 [file Table_1.DOCX]

Supplementary Material

# Supplementary Table 1. The excluded articles and the reasons

| A total of 681 records were identified by searching in English and Chinese databases at first. After 612 records were deleted through screening by title and/or Abstract, 69 full texts remained to be further selected. Of which, 33 articles were removed because of the following reasons: | |
| --- | --- |
| Case report = 1 | 1. Gan, J., Zhou, H., Liu, C., and Fang, L. (2023). PSEN2 and ABCA7 variants causing early-onset preclinical pathological changes in Alzheimer's disease: a case report and literature review. Neurol Sci 44(6), 1987-2001. doi: 10.1007/s10072-023-06602-5. |
| Animal studies = 1 | 1. Dehghan, A., Pinto, R.C., Karaman, I., Huang, J., Durainayagam, B.R., Ghanbari, M., et al. (2022). Metabolome-wide association study on ABCA7 indicates a role of ceramide metabolism in Alzheimer's disease. Proc Natl Acad Sci U S A 119(43), e2206083119. doi: 10.1073/pnas.2206083119. |
| Reviews =1 | 1. Karaca, I., Wagner, H., and Ramirez, A. (2017). [Search for risk genes in Alzheimer's disease]. Nervenarzt 88(7), 744-750. doi: 10.1007/s00115-017-0354-7. |
| Meta-analysis =6 | 1. Bao, J., Wang, X.J., and Mao, Z.F. (2016). Associations Between Genetic Variants in 19p13 and 19q13 Regions and Susceptibility to Alzheimer Disease: A Meta-Analysis. Med Sci Monit 22, 234-243. doi: 10.12659/msm.895622.  2. Zhou, G., Mao, X., Chu, J., Chen, G., Zhao, Q., Wang, L., and Luo, Y. (2017). ATP binding cassette subfamily A member 7 rs3764650 polymorphism and the risk of Alzheimer's disease. Pharmazie 72(7), 425-427. doi: 10.1691/ph.2017.6862.  3. Liu, G., Li, F., Zhang, S., Jiang, Y., Ma, G., Shang, H., et al. (2014). Analyzing large-scale samples confirms the association between the ABCA7 rs3764650 polymorphism and Alzheimer's disease susceptibility. Mol Neurobiol 50(3), 757-764. doi: 10.1007/s12035-014-8670-4.  4. Wang, J., Kong, X.Y., Cong, L.L., Xu, Z.X., Du, J.S., Cong, X.L., et al. (2018). Associations between CD33 rs3865444 and ABCA7 rs3764650 polymorphisms and susceptibility to Alzheimer's disease. J Integr Neurosci 17(4), 313-321. doi: 10.31083/j.jin.2018.04.0408.  5. Ma, F.C., Wang, H.F., Cao, X.P., Tan, C.C., Tan, L., and Yu, J.T. (2018). Meta-Analysis of the Association between Variants in ABCA7 and Alzheimer's Disease. J Alzheimers Dis 63(4), 1261-1267. doi: 10.3233/jad-180107.  6. Almeida, J.F.F., Dos Santos, L.R., Trancozo, M., and de Paula, F. (2018). Updated Meta-Analysis of BIN1, CR1, MS4A6A, CLU, and ABCA7 Variants in Alzheimer's Disease. J Mol Neurosci 64(3), 471-477. doi: 10.1007/s12031-018-1045-y. |
| Not meet the purpose of the meta-analysis = 10 | 1. Allen, M., Zou, F., Chai, H.S., Younkin, C.S., Crook, J., Pankratz, V.S., et al. (2012). Novel late-onset Alzheimer disease loci variants associate with brain gene expression. Neurology 79(3), 221-228. doi: 10.1212/WNL.0b013e3182605801.  2. Chibnik, L.B., Yu, L., Eaton, M.L., Srivastava, G., Schneider, J.A., Kellis, M., et al. (2015). Alzheimer's loci: epigenetic associations and interaction with genetic factors. Ann Clin Transl Neurol 2(6), 636-647. doi: 10.1002/acn3.201.  3. Ghani, M., Pinto, D., Lee, J.H., Grinberg, Y., Sato, C., Moreno, D., et al. (2012). Genome-wide survey of large rare copy number variants in Alzheimer's disease among Caribbean hispanics. G3 (Bethesda) 2(1), 71-78. doi: 10.1534/g3.111.000869.  4. Holton, P., Ryten, M., Nalls, M., Trabzuni, D., Weale, M.E., Hernandez, D., et al. (2013). Initial assessment of the pathogenic mechanisms of the recently identified Alzheimer risk Loci. Ann Hum Genet 77(2), 85-105. doi: 10.1111/ahg.12000.  5. Schott, J.M., Crutch, S.J., Carrasquillo, M.M., Uphill, J., Shakespeare, T.J., Ryan, N.S., et al. (2016). Genetic risk factors for the posterior cortical atrophy variant of Alzheimer's disease. Alzheimers Dement 12(8), 862-871. doi: 10.1016/j.jalz.2016.01.010.  6. Verhaaren, B.F., Vernooij, M.W., Koudstaal, P.J., Uitterlinden, A.G., van Duijn, C.M., Hofman, A., et al. (2013). Alzheimer's disease genes and cognition in the nondemented general population. Biol Psychiatry 73(5), 429-434. doi: 10.1016/j.biopsych.2012.04.009.  7. Ma, F.C., Zong, Y., Wang, H.F., Li, J.Q., Cao, X.P., and Tan, L. (2018). ABCA7 genotype altered Aβ levels in cerebrospinal fluid in Alzheimer's disease without dementia. Ann Transl Med 6(22), 437. doi: 10.21037/atm.2018.07.04.  8. Carrasquillo, M.M., Khan, Q., Murray, M.E., Krishnan, S., Aakre, J., Pankratz, V.S., et al. (2014). Late-onset Alzheimer disease genetic variants in posterior cortical atrophy and posterior AD. Neurology 82(16), 1455-1462. doi: 10.1212/wnl.0000000000000335.  9. Sherva, R., Zhang, R., Sahelijo, N., Jun, G., Anglin, T., Chanfreau, C., et al. (2023). African ancestry GWAS of dementia in a large military cohort identifies significant risk loci. Mol Psychiatry 28(3), 1293-1302. doi: 10.1038/s41380-022-01890-3.  10.Vardarajan, B.N., Ghani, M., Kahn, A., Sheikh, S., Sato, C., Barral, S., et al. (2015). Rare coding mutations identified by sequencing of Alzheimer disease genome-wide association studies loci. Ann Neurol 78(3), 487-498. doi: 10.1002/ana.24466. |
| Just one study of SNP involved=2 | 1. Csaban, D., Illes, A., Renata, T.B., Balicza, P., Pentelenyi, K., Molnar, V., et al. (2022). Genetic landscape of early-onset dementia in Hungary. Neurol Sci 43(9), 5289-5300. doi: 10.1007/s10072-022-06168-8.  2. Zhang, W.W., Jiao, B., Xiao, T.T., Liu, X.X., Liao, X.X., Xiao, X.W., et al. (2020). Association of rare variants in neurodegenerative genes with familial Alzheimer's disease. Ann Clin Transl Neurol 7(10), 1985-1995. doi: 10.1002/acn3.51197. |
| Overlapped data=5 | 1. Allen, M., Lincoln, S.J., Corda, M., Watzlawik, J.O., Carrasquillo, M.M., Reddy, J.S., et al. (2017). ABCA7 loss-of-function variants, expression, and neurologic disease risk. Neurol Genet 3(1), e126. doi: 10.1212/nxg.0000000000000126.  2. Santos, L.R.D., Almeida, J.F.F., Pimassoni, L.H.S., Morelato, R.L., and Paula, F. (2020). The combined risk effect among BIN1, CLU, and APOE genes in Alzheimer's disease. Genet Mol Biol 43(1), e20180320. doi: 10.1590/1678-4685-gmb-2018-0320.  3. Kunkle, B.W., Schmidt, M., Klein, H.U., Naj, A.C., Hamilton-Nelson, K.L., Larson, E.B., et al. (2021). Novel Alzheimer Disease Risk Loci and Pathways in African American Individuals Using the African Genome Resources Panel: A Meta-analysis. JAMA Neurol 78(1), 102-113. doi: 10.1001/jamaneurol.2020.3536.  4. Hohman, T.J., Koran, M.E., and Thornton-Wells, T. (2013). Epistatic genetic effects among Alzheimer's candidate genes. PLoS One 8(11), e80839. doi: 10.1371/journal.pone.0080839.  5. Stage, E., Risacher, S.L., Lane, K.A., Gao, S., Nho, K., Saykin, A.J., and Apostolova, L.G. (2022). Association of the top 20 Alzheimer's disease risk genes with [(18)F]flortaucipir PET. Alzheimers Dement (Amst) 14(1), e12308. doi: 10.1002/dad2.12308. |
| Insufficient data =7 | 1. Beecham, G.W., Hamilton, K., Naj, A.C., Martin, E.R., Huentelman, M., Myers, A.J., et al. (2014). Genome-wide association meta-analysis of neuropathologic features of Alzheimer's disease and related dementias. PLoS Genet 10(9), e1004606. doi: 10.1371/journal.pgen.1004606.  2. Ebbert, M.T., Ridge, P.G., Wilson, A.R., Sharp, A.R., Bailey, M., Norton, M.C., et al. (2014). Population-based analysis of Alzheimer's disease risk alleles implicates genetic interactions. Biol Psychiatry 75(9), 732-737. doi: 10.1016/j.biopsych.2013.07.008.  3. Kamboh, M.I., Demirci, F.Y., Wang, X., Minster, R.L., Carrasquillo, M.M., Pankratz, V.S., et al. (2012). Genome-wide association study of Alzheimer's disease. Transl Psychiatry 2(5), e117. doi: 10.1038/tp.2012.45.  4. Shi, H., Belbin, O., Medway, C., Brown, K., Kalsheker, N., Carrasquillo, M., et al. (2012). Genetic variants influencing human aging from late-onset Alzheimer's disease (LOAD) genome-wide association studies (GWAS). Neurobiol Aging 33(8), 1849.e1845-1818. doi: 10.1016/j.neurobiolaging.2012.02.014.  5. Vasquez, J.B., Fardo, D.W., and Estus, S. (2013). ABCA7 expression is associated with Alzheimer's disease polymorphism and disease status. Neurosci Lett 556, 58-62. doi: 10.1016/j.neulet.2013.09.058.  6. Lambert, J.C., Heath, S., Even, G., Campion, D., Sleegers, K., Hiltunen, M., et al. (2009). Genome-wide association study identifies variants at CLU and CR1 associated with Alzheimer's disease. Nat Genet 41(10), 1094-1099. doi: 10.1038/ng.439.  7. Yang, P., Sun, Y.M., Liu, Z.J., Tao, Q.Q., Li, H.L., Lu, S.J., and Wu, Z.Y. (2013). Association study of ABCA7 and NPC1 polymorphisms with Alzheimer's disease in Chinese Han ethnic population. Psychiatr Genet 23(6), 268. doi: 10.1097/ypg.0000000000000016. |

# Supplementary Table 2. Characteristics of included studies and ABCA7 SNPs for meta-analysis

| **First author**  **(Year)** | **Countries**  **(Populations)** | **Variants** | **Samples Selection/Characteristics** | | **NOS**  **Score** | | **HWE** |
| --- | --- | --- | --- | --- | --- | --- | --- |
|  |  |  | **AD Cases** | **Controls** |  |  |  |
| Harold D 2009 | UK  Germany  America  NHWs | rs3764650 | N = 3332.  Age and gender were described in each cohort.  Diagnostic criteria for AD: NINCDS-ADRDA, DSM-IV, or CERAD. | N = 6358.  Age and gender were described in each cohort. | | 7 | UK: N  Germany: Y  USA: Y |
| Hollingworth P 2011 | America  UK  Iceland  France  NHWs | rs3764650 | N = 11376.  Age and gender were described in each cohort.  Diagnostic criteria for AD: NINCDS-ADRDA, DSM-IV, or CERAD. | N = 22450.  Age and gender were described in each cohort. | | 7 | GERAD2: Y  deCODE: Y  AD-IG: Y  EADI2: Y  Mayo2: Unavailable  CHARGE: Unavailable |
| Naj AC 2011 | America  NHWs | rs3764650  rs3752246 | N (rs3764650) = 10011.  N (rs3752246) = 11171.  Age and gender were described in each cohort.  Diagnostic criteria for AD: NINCDS-ADRDA. | N (rs3764650) = 8949.  N (rs3752246) = 10218.  Age and gender were described in each cohort. | | 7 | Y |
| Logue MW 2011 | America  Mixed populations | rs3764650  rs3752246  rs3764647 | N = 513.  Age and gender were described in each cohort.  Diagnostic criteria for AD: NINCDS-ADRDA. | N = 496.  Age and gender were described in each cohort. | | 9 | Unavailable |
| Chung SJ 2013 | Korea  Asians | rs3764650  rs3752232  rs3752229  rs4147932  rs3752237  rs3752243 | LOAD :N = 290, age: 74.86 ± 9.14, Age at onset: 70.52 ± 9.90, F: 194 (66.9%).  Diagnostic criteria for AD: NINCDS-ADRDA. | N = 554, Age: 64.68±9.26, F: 287 (51.8%). | | 8 | Y |
| Cascorbi I 2013 | Germany NHWs | rs3764650 | N = 71, age: 78.4±10.7, F: 39 (54.9%).  Diagnostic criteria for AD: CERAD. | N = 80, HB, age: 69.5 ± 11.4, F: 33 (40.7%). | | 7 | Y |
| Miyashita A 2013 | Japan  Asians | rs3764650 | LOAD: N = 1008, Age at onset: 73.0 (4.28%), F: 723 (72%).  Diagnostic criteria for AD: NINCDS-ADRDA. | N = 1016, PB, age: 77 ± 5.89, F: 583 (57%). | | 9 | Y |
| Lambert JC 2013 | Austria  Belgium  Finland  Germany  Greece  Hungary  Italy  Spain  Sweden  UK  America  NHWs | rs4147929 | N = 8572.  Age and gender were described in each cohort.  Diagnostic criteria for AD: DSM-III-R and NINCDS-ADRDA. | N = 11312.  Age and gender were described in each cohort. | | 8 | Y |
| Tan L 2013 | China  Asians | rs3764650  rs3752246 | LOAD, N = 612, age: 80.85 ± 7.3, age at onset: 75.60±6.2, F: 321 (52.5%).  Diagnostic criteria for AD: NINCDS-ADRDA. | N = 612, HB, age: 74.93 ± 6.3, F: 287 (46.9%). | | 8 | Y |
| Reitz C 2013 | America  Mixed populations | rs115550680 | LOAD, N = 1968.  Age and gender were described in each cohort.  Diagnostic criteria for AD: NINCDS-ADRDA. | N = 3928 , PB.  Age and gender were described in each cohort. | | 9 | Y |
| Omoumi A 2014 | Canada  NHWs | rs3764650 | N = 569.  No description of age and gender exactly.  Diagnostic criteria for AD: DSM-III-R and NINCDS-ADRDA. | N = 494.  No description of age and gender exactly. | | 7 | Y |
| Liu LH 2014 | China  Asians | rs3764650 | N = 350, age: 72.83 ± 8.51. F: 176 (50.29%).  Diagnostic criteria for AD: NINCDS-ADRDA. | N = 283, age:73.57 ± 7.15, F: 161 (56.89%). | | 8 | Y |
| Liao YC 2014 | China(Taiwan)  Asians | rs3764650 | N = 534, age: 79.6 ± 7.9, F: 247 (46.3%).  Diagnostic criteria for AD: NINCDS-ADRDA. | N = 307, HB, age: 76.2±10.0, F: 96 (30.6%). | | 7 | N |
| Cuyvers E 2015 | Belgium  NHWs | rs3764645  rs4147914  rs4147934  rs4147932  rs3752246  rs4147929  rs3764650 | N = 772, Age at onset: 74.6 ± 8.9, F: 500 (65%).  Diagnostic criteria for AD: NINCDS-ADRDA. | N = 757, PB, age: 73.9 ± 8.0, F: 454 (60%). | | 8 | Unavailable |
| Öznur M 2015 | Turkey  NHWs | rs3764650 | N = 54, age: 77.4, F: 30 (55.6%).  Only mentioned that AD cases were clinically diagnosed, but no specific diagnostic criteria. | N = 57, age: 70.9, F: 37 (64.9%). | | 7 | Y |
| Steinberg S 2015 | America  Finland  Germany  Norway  NHWs | rs113809142  rs200538373 | N = 2365.  No description of age and gender.  Diagnostic criteria for AD: NINCDS-ADRDA. | N = 4316.  No description of age and gender. | | 9 | Unavailable |
| Sassi C 2016 | UK  America  NHWs | rs3752246  rs74176364  rs117187003  rs3752239  rs3764647  rs3752232  rs4147918  rs3764645  rs4147934  rs3745842 | N = 331, Age at onset: 71.66, F: 139 (42%).  Diagnostic criteria for AD: NINCDS-ADRDA. | N = 672, age: 78.15, F: 343 (51%). | | 7 | Y |
| Cukier HN 2016 | America  African Americans | rs115550680 | N = 532.  No description of age and gender.  Diagnostic criteria for AD: NINCDS-ADRDA. | N = 519.  No description of age and gender. | | 7 | Unavailable |
| Yamazaki K 2017 | Japan  Asians | rs3764650 | N = 50, age: 77.7 ± 6.05, F: 39 (78%).  Diagnostic criteria for AD: NIA-AA. | N = 50, age: 76.3 ± 6.02, F: 39 (78%). | | 8 | Y |
| Li H 2017 | China  Asians | rs3764650 | N = 118, age: 71.81 ± 9.66, F: 43 (36.4%).  Diagnostic criteria for AD: NINCDS-ADRDA. | N = 120, age: 70.45 ± 9.52, F: 52 (43.3%). | | 8 | N |
| Dos Santos LR 2017 | Brazil  Mixed populations | rs3764650 | LOAD, N = 77, F: 54 (68.4%).  No description of age.  Diagnostic criteria for AD: NINCDS-ADRDA. | N = 144, F: 106 (73.1%).  No description of age. | | 8 | Y |
| Moreno DJ 2017 | Columbia  Mixed populations | rs3764650 rs3752246  rs3752229 | LOAD, N = 280, age: 75.5 ± 7.23, F: 213 (76.1%).  Diagnostic criteria for AD: DSM-IV. | N = 357, age: 71.04±7.08, F: 264 (73.9%). | | 8 | Y |
| Patel T 2018 | UK  Mixed populations | rs3764645  rs3752234  rs3752237  rs4147915  rs3752232  rs3764647  rs3752239  rs74176364  rs3745842  rs3752246  rs117187003  rs4147918  rs4147934 | LOAD, N = 132, age at onset >65 , F: 70 (53.0%).  Diagnostic criteria for AD: CREAD and NIA-AA. | N = 53, F: 27 (50.9%). | | 7 | Y |
| Liu J 2018 | China  Asians | rs3764650  rs3752246  rs3752242  rs4147929 | N = 118, age: 74.6 ± 7.85, F: 43 (36.4%).  Diagnostic criteria for AD: DSM-IV-TR and NIA-AA. | N = 139, age: 72.7±6.21, F: 64 (46.0%). | | 9 | Y |
| Moreno-Grau S 2018 | Spain  NHWs | rs4147929 | N = 1500, age: 82.1 ± 7.9, F: 1053 (70.2%).  Diagnostic criteria for AD: DSM-IV and NINCDS-ADRDA. | N = 2494, age: 54.1 ±11.6, F: 1604 (64.3%). | | 7 | Y |
| Zhang JR 2018 | China  Asians | rs3752246  rs3764650 | LOAD, N = 300, age: 73.394 ±9.475, F: 152 (51%).  Diagnostic criteria for AD: DSM-IV. | N = 508, age: 64.873 ±5.173, F: 245 (48%). | | 8 | Y |
| Kjeldsen EW 2018 | Denmark NHWs | rs4147929 | N = 962.  No description of age and gender.  Diagnostic criteria for AD: NINCDS-ADRDA. | N = 102198.  No description of age and gender. | | 7 | Y |
| Fehér Á 2019 | Hungary  NHWs | rs3752246 | LOAD, N = 416, age: 74.8 ± 6.7, F: 272 (65.4%).  Diagnostic criteria for AD: NINCDS-ADRDA. | N = 302., age: 74.2 ± 7.3. F: 193 (64.0%). | | 8 | Y |
| Talebi M 2019 | Iran  NHWs | rs3764650  rs4147929 | LOAD, n = 110, age＞65 ,F: 63 (57.27%).  Diagnostic criteria for AD: NINCDS-ADRDA. | N=88, PB, age＞65, F: 56 (63.63%). | | 9 | N |
| Nazaketi N 2020 | China  Mixed populations | rs3764650 | N = 131, age= 75.2± 7.6, F: 75 (57.3%).  Diagnostic criteria for AD: DSM-IV. | N = 128. HB, age: 74.4± 7.9. F: 70 (54.7%). | | 7 | Y |
| Ren M 2020 | China  Mixed populations | rs3764650  rs4147929 | N = 745, age≥55, F: 379 (50.9%).  Diagnostic criteria for AD: NINCDS-ADRDA. | N = 433, age≥55, F: 298 (68.8%). | | 7 | Y |
| Abd Elrahman HG 2020 | Egypt  NHWs | rs3764650 | N = 100, age: 70.7 ± 7.5, F:55 (55%).  Diagnostic criteria for AD: DSM-IV. | N = 100, PB, age: 65.9 ± 5.02, F: 76 (76%). | | 8 | Y |
| Hou M 2021 | China  Asians | rs3764650 | N = 30, age: 71.9 ± 6.9, F:13 (43.3%).  Diagnostic criteria for AD: NINCDS-ADRDA and DSM-III. | N = 47, age: 71.1 ± 6.7, F: 25 (53.2%). | | 7 | Y |
| Wang L 2022 | China  Asians | rs3764650  rs4147929 | N = 246, age: 71.26 ± 8.46, F: 137 (55.7%).  Diagnostic criteria for AD: NINCDS-ADRDA. | N = 244, HB, age: 71.10 ± 8.31, F: 137 (56.1%). | | 9 | Y |
| Campbell AS 2022 | America  NHWs | rs113809142  rs200538373 | N = 2495, age: 79.63 ± 8.25, F: 1491 (60%).  Diagnostic criteria for AD: NINCDS-ADRDA. | N = 2858, age: 81.54 ± 6.25, F: 1567 (55%). | | 7 | Y |
| Jiao B 2022 | China  Asians | rs3752246  rs3752229  rs4147914  rs4147934  rs3764645  rs3752243  rs3764647  rs4147915  rs3752232 | N = 1192, age: 64 ± 18, F: 717 (60.2%).  Diagnostic criteria for AD: NIA-AA. | N = 2412, PB, age: 65 ± 10, F: 1255 (52.0%). | | 8 | Y |

Notes: AD: Alzheimer’s disease; LOAD: late-onset AD; HWE: Hardy-Weinberg equilibrium; NHWs: non-Hispanic Whites; Y: the controls being HWE; N: the controls deviating from HWE; NOS: Newcastle-Ottawa Scale; PB: population-based; HB: hospital-based; F: female; Unavailable: The HWE analysis is not performed due to allele frequencies alone, or HWE is not mentioned in the original text; NINCDS-ADRDA: National Institute of Neurological and Communicative Disorders and Stroke - Alzheimer's Disease and Related Disorders Association Criteria; DSM-IV: Diagnostic and Statistical Manual of Mental Disorders, Fourth Edition; CERAD: Consortium to Establish a Registry for Alzheimer's Disease; DSM-III-R: Diagnostic and Statistical Manual of Mental Disorders, Third Edition, Revised; NIA-AA: National Institute on Aging - Alzheimer's Association.

# Supplementary Table 3. Summary of Meta-analysis results

| **ABCA7 Polymorphisms** | **Groups** | **AD Cases** | | |  | **controls** | | |  | **Comparison models**  **OR[95%CI], *P* value (*P*_FDR_)** | | |
| --- | --- | --- | --- | --- | --- | --- | --- | --- | --- | --- | --- | --- |
| rs3752246 |  | CC | CG | GG |  | CC | CG | GG |  | A | D | R |
|  | Combined Population | 806 | 553 | 118 |  | 1028 | 548 | 149 |  | 1.17[1.13, 1.22],  <0.0001*（0.0003）* | 0.89[0.77, 1.03],  0.11（0.165） | 0.95[0.55, 1.66],  0.86（0.86） |
|  | Asians | 314 | 287 | 71 |  | 323 | 283 | 66 |  | 1.24[1.14, 1.35],  <0.0001*（0.0003）* | 0.95[0.76,1.17],  0.62（0.65） | 1.08[0.76,1.55],  0.65（0.65） |
|  | NHWs | 463 | 250 | 34 |  | 659 | 242 | 73 |  | 1.16[1.10, 1.22],  <0.0001*（0.0003）* | 0.85[0.69, 1.05],  0.13（0.195） | 0.74[0.22, 2.52],  0.63（0.63） |
|  | Late-onset AD | 516 | 423 | 89 |  | 481 | 360 | 73 |  | 1.15[1.10, 1.21],  <0.0001*（0.0003）* | 0.87[0.73, 1.04],  0.13（0.195） | 1.16 [0.84, 1.61],  0.37（0.37） |
| rs3764650 |  | TT | TG | GG |  | TT | TG | GG |  | A | D | R |
|  | Combined Population | 4958 | 1967 | 334 |  | 7416 | 2217 | 274 |  | 1.15[1.09, 1.21],  <0.0001*(0.0003)* | 0.96[0.84, 1.07],  0.36（0.36） | 1.19 [0.86, 1.66],  0.29（0.36） |
|  | Asians | 1115 | 933 | 264 |  | 980 | 790 | 190 |  | 1.10 [1.03, 1.17],  0.003*（0.009）* | 0.94[0.83, 1.06],  0.31（0.31） | 1.39[0.90, 2.15],  0.13（0.195） |
|  | NHWs | 3425 | 811 | 42 |  | 6048 | 1188 | 41 |  | 1.19[1.11, 1.28]  <0.0001*（0.0003）* | 0.89[0.74, 1.08],  0.24（0.24） | 1.78[1.16, 2.75],  0.009*（0.0135）* |
|  | Late-onset AD | 922 | 379 | 67 |  | 923 | 346 | 69 |  | 1.17[1.11, 1.23],  <0.0001*（0.0003）* | 0.86[0.72, 1.02],  0.09（0.135） | 0.99[0.69, 1.41],  0.95（0.95） |
| rs4147929 |  | GG | GA | AA |  | GG | GA | AA |  | A | D | R |
|  | Combined Population | 1267 | 781 | 133 |  | 73143 | 27408 | 2693 |  | 1.11[1.02, 1.22],  0.017*（0.051） | 0.97[0.77, 1.21],  0.76（0.76） | 0.51[0.18, 1.43],  0.20（0.3） |
|  | Asians | 262 | 226 | 57 |  | 266 | 87 | 141 |  | 1.21[1.00, 1.47],  0.049*（0.09） | 0.79[0.62, 1.01],  0.06（0.09） | 0.41[0.11, 1.52],  0.18（0.18） |
|  | NHWs | 705 | 329 | 38 |  | 72636 | 27140 | 2510 |  | 1.16[1.11, 1.21],  <0.0001*（0.0003）* | 1.15[0.61, 2.17],  0.66（0.69） | 0.63[0.07, 6.12],  0.69（0.69） |
| rs3764647 |  | AA | AG | GG |  | AA | AG | GG |  | A | D | R |
|  | Combined Population | - | - | - |  | - | - | - |  | 0.97[0.66, 1.42],  0.871 | - | - |
| rs3752229 |  | AA | AG | GG |  | AA | AG | GG |  | A | D | R |
|  | Combined Population | - | - | - |  | - | - | - |  | 1.14[0.91,1.44],  0.25 | - | - |
|  | Asians | - | - | - | - | - | - | - | - | 1.12[0.85,1.47],  0.44 | - | - |
|  | Late-onset AD | - | - | - | - | - | - | - | - | 0.99[0.81,1.20],  0.91 | - | - |
| rs3752232 |  | AA | AG | GG |  | AA | AG | GG |  | A | D | R |
|  | Combined Population |  |  |  |  | - | - | - |  | 0.82[0.70, 0.95],  0.01* | - | - |
|  | Asians |  |  |  |  | - | - | - |  | 0.82[0.69, 0.96],  0.01* | - | - |
|  | Late-onset AD |  |  |  |  | - | - | - |  | 1.02[0.71, 1.43],  0.93 | - | - |
| rs3752237 |  | GG | GA | AA |  | GG | GA | AA |  | A | D | R |
|  | Combined Population | - | - | - |  | - | - | - |  | 1.92[0.93,3.90],  0.08 | - | - |
| rs3752243 |  | AA | AG | GG |  | AA | AG | GG |  | A | D | R |
|  | Combined Population | - | - | - |  | - | - | - |  | 0.84[0.76, 0.92],  0.0001* | - | - |
| rs3764645 |  | GG | GA | AA |  | GG | GA | AA |  | A | D | R |
|  | Combined Population | - | - | - |  | - | - | - |  | 0.85[0.79, 0.92],  <0.0001* | - | - |
|  | NHWs | - | - | - |  | - | - | - |  | 0.90[0.81, 1.01],  0.07 | - | - |
| rs4147932 |  | CC | CT | TT |  | CC | CT | TT |  | A | D | R |
|  | Combined Population | - | - | - |  | - | - | - |  | 0.96[0.65, 1.41],  0.83 | - | - |
| rs4147934 |  | TT | TG | GG |  | TT | TG | GG |  | A | D | R |
|  | Combined Population | - | - | - |  | - | - | - |  | 1.07[0.83, 1.38],  0.62 | - | - |
|  | NHWs | - | - | - |  | - | - | - |  | 1.21[1.07, 1.37],  0.002* | - | - |
| rs113809142 |  | TT | TG | GG |  | TT | TG | GG |  | A | D | R |
|  | Combined Population | 2480 | 3 | 0 |  | 2844 | 1 | 0 |  | 2.47[0.57,10.58],  0.22 | - | - |
| rs200538373 |  | GG | GC | CC |  | GG | GC | CC |  | A | D | R |
|  | Combined Population | 2423 | 40 | 0 |  | 2803 | 20 | 0 |  | 1.71[1.20, 2.44],  0.003* | - | - |
| rs3745842 |  | GG | GA | AA |  | GG | GA | AA |  | A | D | R |
|  | Combined Population | - | - | - |  | - | - | - |  | 0.92[0.48, 1.74],  0.79 | - | - |
| rs3752239 |  | AA | AC | CC |  | AA | AC | CC |  | A | D | R |
|  | Combined Population | - | - | - |  | - | - | - |  | 0.83[0.48, 1.44],  0.50 | - | - |
| rs4147914 |  | GG | GA | AA |  | GG | GA | AA |  | A | D | R |
|  | Combined Population | - | - | - |  | - | - | - |  | 1.28[1.17, 1.40],  <0.0001* | - | - |
| rs4147915 |  | CC | CA | AA |  | CC | CA | AA |  | A | D | R |
|  | Combined Population | - | - | - |  | - | - | - |  | 0.85[0.77, 0.94],  0.001* | - | - |
| rs4147918 |  | AA | AG | GG |  | AA | AG | GG |  | A | D | R |
|  | Combined Population | - | - | - |  | - | - | - |  | 0.90 [0.55, 1.47],  0.68 | - | - |
| rs74176364 |  | GG | GA | AA |  | GG | GA | AA |  | A | D | R |
|  | Combined Population | - | - | - |  | - | - | - |  | 0.32[0.10, 1.06],  0.06 | - | - |
| rs115550680 |  | GG | GA | AA |  | GG | GA | AA |  | A | D | R |
|  | Combined Population | - | - | - |  | - | - | - |  | 1.84[1.55, 2.17],  <0.0001* | - | - |
| rs117187003 |  | GG | GA | AA |  | GG | GA | AA |  | A | D | R |
|  | Combined Population | - | - | - |  | - | - | - |  | 0.89[0.07, 11.12],  0.93 | - | - |

Notes: AD: Alzheimer's disease; A: Allelic model; D: Dominant model; R: Recessive model; *P*_FDR_: *P* value corrected by FDR method;

NHWs: non-Hispanic Whites; *: significant statistically.

# Supplementary Table 4. Results of Linkage disequilibrium analysis

| SNPs^*^ | Population | N | R^2^ | D’ |
| --- | --- | --- | --- | --- |
| rs3764650  rs3752246 | EUR | 503 | 0.2204 | 0.6623 |
|  | EAS | 504 | 0.6401 | 0.8494 |
| rs3764650  rs4147929 | EUR | 503 | 0.2135 | 0.6412 |
|  | EAS | 504 | 0.63 | 0.8445 |
| rs3764650  rs3752232 | EUR | 503 | 0.0044 | 1 |
|  | EAS | 504 | 0.049 | 1 |
| rs3764650  rs3752243 | EUR | 503 | 0.079 | 0.9554 |
|  | EAS | 504 | 0.1834 | 0.7845 |
| rs3764650  rs3764645 | EUR | 503 | 0.091 | 0.9789 |
|  | EAS | 504 | 0.3316 | 0.944 |
| rs3764650  rs4147934 | EUR | 503 | 0.1076 | 0.604 |
|  | EAS | 504 | 0.2538 | 0.8308 |
| rs3764650  rs200538373 | EUR | 503 | 0.0006 | 1 |
|  | EAS | 504 | NA | NA |
| rs3764650  rs4147914 | EUR | 503 | 0.1076 | 0.604 |
|  | EAS | 504 | 0.2538 | 0.8308 |
| rs3764650  rs4147915 | EUR | 503 | 0.0141 | 0.9235 |
|  | EAS | 504 | 0.2753 | 0.9836 |
| rs3764650  rs115550680 | EUR | 503 | NA | NA |
|  | EAS | 504 | NA | NA |
| rs3752246  rs4147929 | EUR | 503 | 0.9679 | 1 |
|  | EAS | 504 | 0.9613 | 0.9826 |
| rs3752246  rs3752232 | EUR | 503 | 0.0087 | 1 |
|  | EAS | 504 | 0.0316 | 0.7557 |
| rs3752246  rs3752243 | EUR | 503 | 0.1637 | 0.9753 |
|  | EAS | 504 | 0.2657 | 0.8894 |
| rs3752246  rs3764645 | EUR | 503 | 0.1674 | 0.9413 |
|  | EAS | 504 | 0.2813 | 0.8189 |
| rs3752246  rs4147934 | EUR | 503 | 0.5366 | 0.956 |
|  | EAS | 504 | 0.4037 | 0.987 |
| rs3752246  rs200538373 | EUR | 503 | 0.0012 | 1 |
|  | EAS | 504 | NA | NA |
| rs3752246  rs4147914 | EUR | 503 | 0.0952 | 0.3348 |
|  | EAS | 504 | 0.493 | 0.7676 |
| rs3752246  rs4147915 | EUR | 503 | 0.0276 | 0.915 |
|  | EAS | 504 | 0.2307 | 0.8482 |
| rs3752246  rs115550680 | EUR | 503 | NA | NA |
|  | EAS | 504 | NA | NA |
| rs4147929  rs3752232 | EUR | 503 | 0.0084 | 1 |
|  | EAS | 504 | 0.0318 | 0.7564 |
| rs4147929  rs3752243 | EUR | 503 | 0.2455 | 0.8529 |
|  | EAS | 504 | 0.1666 | 1 |
| rs4147929  rs3764645 | EUR | 503 | 0.1615 | 0.9398 |
|  | EAS | 504 | 0.274 | 0.8065 |
| rs4147929  rs4147934 | EUR | 503 | 0.5513 | 0.985 |
|  | EAS | 504 | 0.4162 | 1 |
| rs4147929  rs200538373 | EUR | 503 | 0.0011 | 1 |
|  | EAS | 504 | NA | NA |
| rs4147929  rs4147914 | EUR | 503 | 0.0922 | 0.3241 |
|  | EAS | 504 | 0.4782 | 0.7544 |
| rs4147929  rs4147915 | EUR | 503 | 0.0266 | 0.9128 |
|  | EAS | 504 | 0.2157 | 0.8183 |
| rs4147929  rs115550680 | EUR | 503 | NA | NA |
|  | EAS | 504 | NA | NA |
| rs3752232  rs3752243 | EUR | 503 | 0.0273 | 1 |
|  | EAS | 504 | 0.0593 | 0.9718 |
| rs3752232  rs3764645 | EUR | 503 | 0.0299 | 1 |
|  | EAS | 504 | 0.0784 | 1 |
| rs3752232  rs4147934 | EUR | 503 | 0.0859 | 0.9611 |
|  | EAS | 504 | 0.0775 | 1 |
| rs3752232  rs200538373 | EUR | 503 | 0.0002 | 1 |
|  | EAS | 504 | NA | NA |
| rs3752232  rs4147914 | EUR | 503 | 0.0074 | 1 |
|  | EAS | 504 | 0.0299 | 0.6724 |
| rs3752232  rs4147915 | EUR | 503 | 0.0052 | 1 |
|  | EAS | 504 | 0.0381 | 0.7971 |
| rs3752232  rs115550680 | EUR | 503 | NA | NA |
|  | EAS | 504 | NA | NA |
| rs3752243  rs3764645 | EUR | 503 | 0.0202 | 0.1848 |
|  | EAS | 504 | 0.3435 | 0.6549 |
| rs3752243  rs4147934 | EUR | 503 | 0.0776 | 0.5145 |
|  | EAS | 504 | 0.4429 | 0.7391 |
| rs3752243  rs200538373 | EUR | 503 | 0.0037 | 1 |
|  | EAS | 504 | NA | NA |
| rs3752243  rs4147914 | EUR | 503 | 0.0034 | 0.1516 |
|  | EAS | 504 | 0.1026 | 0.5054 |
| rs3752243  rs4147915 | EUR | 503 | 0.0395 | 0.4545 |
|  | EAS | 504 | 0.4245 | 0.6669 |
| rs3752243  rs115550680 | EUR | 503 | NA | NA |
|  | EAS | 504 | NA | NA |
| rs3764645  rs4147934 | EUR | 503 | 0.1077 | 0.5784 |
|  | EAS | 504 | 0.6862 | 0.8334 |
| rs3764645  rs200538373 | EUR | 503 | 0.0062 | 1 |
|  | EAS | 504 | NA | NA |
| rs3764645  rs4147914 | EUR | 503 | 0.0252 | 0.3963 |
|  | EAS | 504 | 0.3818 | 0.8728 |
| rs3764645  rs4147915 | EUR | 503 | 0.1644 | 0.9709 |
|  | EAS | 504 | 0.7008 | 0.9574 |
| rs3764645  rs115550680 | EUR | 503 | NA | NA |
|  | EAS | 504 | NA | NA |
| rs4147934  rs200538373 | EUR | 503 | 0.002 | 1 |
|  | EAS | 504 | NA | NA |
| rs4147934  rs4147914 | EUR | 503 | 0.2204 | 0.6649 |
|  | EAS | 504 | 0.3003 | 0.7787 |
| rs4147934  rs4147915 | EUR | 503 | 0.0441 | 0.8869 |
|  | EAS | 504 | 0.5706 | 0.8586 |
| rs4147934  rs115550680 | EUR | 503 | NA | NA |
|  | EAS | 504 | NA | NA |
| rs200538373  rs4147914 | EUR | 503 | 0.001 | 1 |
|  | EAS | 504 | NA | NA |
| rs200538373  rs4147915 | EUR | 503 | 0.0007 | 1 |
|  | EAS | 504 | NA | NA |
| rs200538373  rs115550680 | EUR | 503 | NA | NA |
|  | EAS | 504 | NA | NA |
| rs4147914  rs4147915 | EUR | 503 | 0.028 | 1 |
|  | EAS | 504 | 0.3833 | 1 |
| rs4147914  rs115550680 | EUR | 503 | NA | NA |
|  | EAS | 504 | NA | NA |
| rs4147915  rs115550680 | EUR | 503 | NA | NA |
|  | EAS | 504 | NA | NA |

Notes: ^*^: The SNP in ABCA7 is in linkage disequilibrium with another ABCA7 SNP. EUR: European (Include the Utah Residents from North and West Europe, Toscani in Italia, Finnish in Finland,and British in England and Scotland). EAS: East Asian (Include the Han Chinese in Beijing, China, Japanese in Tokyo, Japan, Southern Han Chinese, Chinese Dai in Xishuangbanna, China, and the Kinh in Ho Chi Minh City, Vietnam). NA: Not available.
